# Peptide Transporters in the Primary Gastrointestinal Tract of Pre-Feeding Mozambique Tilapia Larva

**DOI:** 10.3389/fphys.2019.00808

**Published:** 2019-07-05

**Authors:** Pazit Con, Tali Nitzan, Tatiana Slosman, Sheenan Harpaz, Avner Cnaani

**Affiliations:** ^1^ Department of Poultry and Aquaculture, Institute of Animal Science, Agricultural Research Organization, Rishon LeZion, Israel; ^2^ Department of Animal Sciences, Robert H. Smith Faculty of Agriculture, Food and Environment, The Hebrew University of Jerusalem, Rehovot, Israel

**Keywords:** PepT1, PepT2, larvae, oreochromis, gastrointestinal tract

## Abstract

Fish larvae differ greatly from the adult form in their morphology and organ functionality. The functionality of the gastrointestinal tract depends on the expression of various pumps, transporters, and channels responsible for feed digestion and nutrients absorption. During the larval period, the gastrointestinal tract develops from a simple closed tube, into its complex form with differentiated segments, crypts and villi, as found in the adult. In this study, we characterized the expression of three peptide transporters (PepT1a, PepT1b, and PepT2) in the gastrointestinal tract of Mozambique tilapia (*Oreochromis mossambicus*) larvae along 12 days of development, from pre-hatching to the completion of yolk sac absorption. Gene expression analysis revealed differential and complimentary time-dependent expression of the PepT1 variants and PepT2 along the larval development period. Immunofluorescence analysis showed differential protein localization of the three peptide transporters (PepTs) along the gastrointestinal tract, in a similar pattern to the adult. In addition, PepT1a was localized in mucosal cells in the larvae esophagus, in much higher abundance than in the adults. The results of this study demonstrate specialization of intestinal sections and absorbance potential of the enterocytes prior to the onset of active exogenous feeding, thus pointing to an uncharacterized function and role of the gastrointestinal tract and its transporters during the larval period.

## Introduction

The development of the gastrointestinal tract during larval stages has been studied in many fish species, such as European sea bass (*Dicentrarchus labrax*) (García Hernández et al., 2001; Sucré et al., 2009), Senegalese sole (*Solea senegalensis*) (Ribeiro et al., 1999), Mozambique tilapia (*Oreochromis mossambicus*) (Lo and Weng, 2006), Nile tilapia (*Oreochromis niloticus*) (Tengjaroenkul et al., 2000, 2002), gilthead seabream (*Sparus aurata*) (Sarasquete et al., 1995; Elbal et al., 2004; Mata-Sotres et al., 2016), Atlantic cod (*Gadus morhua*) (Kjorsvik et al., 1991), California halibut (*Paralichthys californicus*) (Gisbert et al., 2004), and Summer flounder (*Paralichthys dentatus*) (Bisbal and Bengtson, 1995). In the early years, studies focused mostly on morphological description, while in recent years, studies have explored gene expression and regulation. While many different species have been studied, there are great species-specific differences in feeding and digestive ontogeny even within the same family (Rønnestad et al., 2013). The zebrafish (*Danio rerio*) has been established as a model organism for various developmental studies. However, when addressing feeding and nutritional physiology, this species is not an ideal model (Ribas and Piferrer, 2014). Unlike most teleost species, the zebrafish lack a stomach, which changes the digestion and absorption processes that occur during feeding. Hence, there is a need to explore and advance our knowledge in other species.

Tilapia is an important group in the cichlid family with increasing research interest in its physiology, genetics, and regulatory processes (Kocher, 2004; Yan et al., 2013; Sacchi et al., 2014). Fujimura and Okada (2007) documented the developmental stages of the Nile tilapia and compared them to the zebrafish larvae development. Although they found some similarity along this period, there are great differences in the time frame of different physiological aspects. For example, while the differentiation of the unpaired fins and the pectoral fins occurs at 22–34 days post fertilization (dpf) in zebrafish, in the Nile tilapia, it was recorded before 10 dpf. By contrast, the yolk sac absorption period is twice as long in the Nile tilapia than in the zebrafish (Fujimura and Okada, 2007). The yolk sac is the main nutrient and energy source for the developing larva before the onset of exogenous feeding. Therefore, this disproportion between growth and yolk utilization raises the need to characterize the development of the tilapia gastrointestinal tract. Moreover, the slow rate of yolk absorption may correspond to slower gastrointestinal development and thus delay digestion and absorption of exogenous feed.

Protein is an important nutrient in fish diets, as it supports energetic supply for physiological processes and growth, in addition to tissue and protein construction (Sire and Vernier, 1992; Dolomatov et al., 2011). In addition to being an energetic source and building blocks for proteins, amino acids also play an important role in many physiological processes such as signaling and gene expression (Wu, 2010). During ontogeny, the yolk is the larvae source for protein (Kamler, 2008). Exogenous feeding is considered a cue for the gastrointestinal development. In the Nile tilapia, exogenous feeding was reported to begin around 12–13 days post fertilization and was coined as “Early Juvenile” period (Fujimura and Okada, 2007). Starved Nile tilapia larvae showed delayed development of the digestive system (Fabillo et al., 2006). However, several studies on Nile and Mozambique tilapia examined the expression and activity of gastrointestinal enzymes, and detected the presence and activity of these enzymes prior to exogenous feeding (Tengjaroenkul et al., 2002; Lo and Weng, 2006). These phenomena of digestion and absorption-related genes expressed prior to exogenous feeding were also recorded for the peptide transporter 1 (PepT1) in zebrafish (Verri et al., 2003). These findings raise questions as to the specific expression period of the peptide transporters (PepTs) in tilapia larval stages.

PepTs are the only known absorption system for small peptides in the intestine, which result from protein digestion and break down, along with free amino acids (FAA). PepTs are solute carriers-proton-dependent transporters, members of the POT family and coded by the *slc15a* genes. These transporters are mainly known for their important role in di- and tri-peptides absorption into the enterocytes – the epithelial cells of the intestine. In mammalians, two peptide transporters have been found, PepT1 and PepT2, but in fish, there are three transporters, with two PepT1 paralogs (Gonçalves et al., 2007; Romano et al., 2014; Con et al., 2017). All three transporters have been found to be expressed in the tilapia intestine, with differential expression along the intestine, depending on the intestinal segments, environmental factors, and nutrient availability in the intestinal lumen (Huang et al., 2015; Con et al., 2017).

In recent years, there has been increased interest and research on PepTs’ participation in protein absorption in fish under different environmental and dietary conditions (Hakim et al., 2009; Bakke et al., 2010b; Bucking and Schulte, 2012; Koven and Schulte, 2012; Verri et al., 2016; Con et al., 2017; Orozco et al., 2017; Chourasia et al., 2018; Hallali et al., 2018; Kokou et al., 2019). However, there are few studies addressing these transporters at the larval and juvenile stages. As these transporters were shown to have an important role in protein absorption, the aim of this study was to characterize them in the early stages of the Mozambique tilapia gastrointestinal development.

## Materials and Methods

### Animals

Mozambique tilapia fish used in this study were derived from a stock maintained at the aquaculture facility of the Agricultural Research Organization (ARO). This stock originated from Natal, South Africa, and was brought to Israel in the 1970s.

### Ethics

This study was approved by the Agricultural Research Organization Committee for Ethics in Experimental Animal Use, and was carried out in compliance with the current laws governing biological research in Israel (Approval number: IL-650/15).

### Tissue Distribution

Tissue samples of gills, esophagus, stomach, liver, spleen, muscle, heart, intestine, kidney, skin, brain, pituitary, and fat were taken from four males Mozambique tilapia (54 ± 3 g). The intestine from each fish was divided into three segments; anterior intestine (AI), middle intestine (MI), and posterior intestine (PI). The tissues were kept in 1 ml of RNA-save buffer (Biological Industries, Mishmar Haemek) at −20°C until RNA extraction procedure.

### Larvae Sampling

Breeding families consisting of one male and 4–6 females, in 200 L aquaria, were constantly monitored to observe spawning. Two days after spawning and fertilization (2 dpf), eggs were removed from the females’ buccal cavities and transferred to hatching jars. Each experiment was conducted on full-sibs from a single spawn.

In order to determine the main tissues expressing the peptide transporters in the larvae, 60 larvae (full sibs from a single spawn) were sampled at 9 dpf and dissected using microsurgery under binocular. The yolk sac was removed and the gastrointestinal tract was separated from the larvae body. Tissue samples (GI tract and Larvae body) from 10 larvae were pooled (six replicas per tissue), and stored in 1 ml of RNA-save buffer at −20°C until RNA extraction procedure.

In order to track the expression of the PepT transcripts along the embryonic period, six embryos from an additional spawn were sampled daily, commencing from 3 dpf to 14 dpf. Each larva served as an individual biological replica. The larvae were kept in 1 ml of RNA-save buffer at −20°C until RNA extraction procedure.

For immunofluorescence staining, a second time-course experiment was conducted. Larvae from a single spawn were sampled each day, between 6 to14 dpf. The larvae were fixed in 4% PFA for 10 min and then dried on paper. The yolk sac was removed using microsurgery under binocular while making sure that the gastrointestinal tract remained untouched. Following the yolk sac removal, the larvae were incubated in 4% PFA for 24 h at 4°C, followed by two washes in PBS, 50% ethanol, and stored in 70% ethanol at 4°C. The larvae were then dehydrated through a series of graded ethanol baths to displace water (1 h in 70, 96, and 100% of ethanol, followed by two Xylen baths for 1 h). Samples were then embedded in paraffin and 5-μm sections were cut using a microtome and placed on microscope slides. The slides were incubated overnight on a 39°C heated plate and were stored at 4°C until staining.

### RNA Extraction and cDNA Synthesis

Total RNA was extracted using Trizol reagent, purified from DNA contamination using TURBO DNA-free Kit (Ambion), quantified with Nano-Drop spectrophotometer (Thermo Scientific), and then reversed transcribed into cDNA using Verso cDNA Synthesis Kit (Thermo Scientific).

### Quantitative Real-Time PCR Analysis (qPCR)

For each gene, forward and reverse primers for qPCR analysis (Table 1) were tested in all samples using a PCR reaction. elongation factor 1 (EF-1), GAPDH, and β-actin genes were used as reference genes. Geometric average was calculated for all reference genes, and this value was used for the relative expression calculation. qPCR reactions were conducted using Fast SYBR™ Green Master Mix on StepOnePlus Real time PCR system (Applied Biosystems). For each set of primers, sequential 1:4 dilutions of cDNA mix were used to create standard curves to determine reaction efficiency, slopes and template dilution. The reaction’s efficiency was confirmed to be in the range of 92.4–105%. The data obtained from the real time PCR were analyzed using the ∆∆Ct method.

**Table 1:** Primers for quantitative real-time PCR.

Gene	GeneBank	Forward	Reverse
*slc15a1a*	KX034112.1	CCAAGCCAGAACAAGGTAACA	GGCTCAATTAGTCCCAAGTCC
*slc15a2*	KX034111.1	CTGCGAACGCTTCTCCTACT	CGCTGAAAGCATGGTAGACA
*slc15a1b*	KX034110.1	TAAAACCCTGCCTGACTTCC	AATCCTCATTAGCCCCAAAA
gapdh	XM_003452690	GGCATCGTGGAAGGTCTCAT	CATTTTACCAGAGGGCCCGT
Beta actin	XM_003443127	CCACCCAAAGTTCAGCCATG	ACGATGGAGGGGAAGACAG
ef1	XM_003458541	TCAACGCTCAGGTCATCATC	ACGGTCGATCTTCTCAACCA

### Immunofluorescence Staining

Tissues sections slides from larvae and adults were stained according to the protocol described by Con et al. (2017). Briefly, slides were prepared for immunostaining using a series of washes with Xylen, decreasing ethanol concentration and PBS-T 0.05% buffer. Antigen retrieval was performed using citrate buffer (1.8 mM citric acid and 8.2 mM sodium citrate) heated to 100°C for 10 min followed by three washes with PBS buffer. Slides were blocked for 1 h at room temperature with blocking solution (1% NGS, 1% BSA in PBS-T 0.05%), followed by 1-h incubation at room temperature with primary antibody solution (1:200 dilution in blocking solution). The slides were washed three times in PBS-T 0.05% and incubated with the secondary antibody solution (goat α rabbit- Cy3 diluted 1:200 in blocking solution) for 1 h at room temperature in the dark. Following incubation, the slides were washed again with PBS-T 0.05% in the dark and stained with 2.85 μM DAPI solution. Following a short rinse in PBS, the slides were covered with a cover glass and the stained sections were examined using a confocal microscope.

### Statistical Analysis

Statistical analyses were conducted separately, for each experiment and each expressed gene, using one-way analysis of variance (ANOVA). *Post hoc* comparisons among groups were performed using the Tukey-Kramer HSD for tissue distribution and larvae time-course real time results, and Student’s *t*-test for the ratio between the GI tract and the larvae body (*α* = 0.05). Data are presented as means ± SEM.

## Results

**Figure 1:**
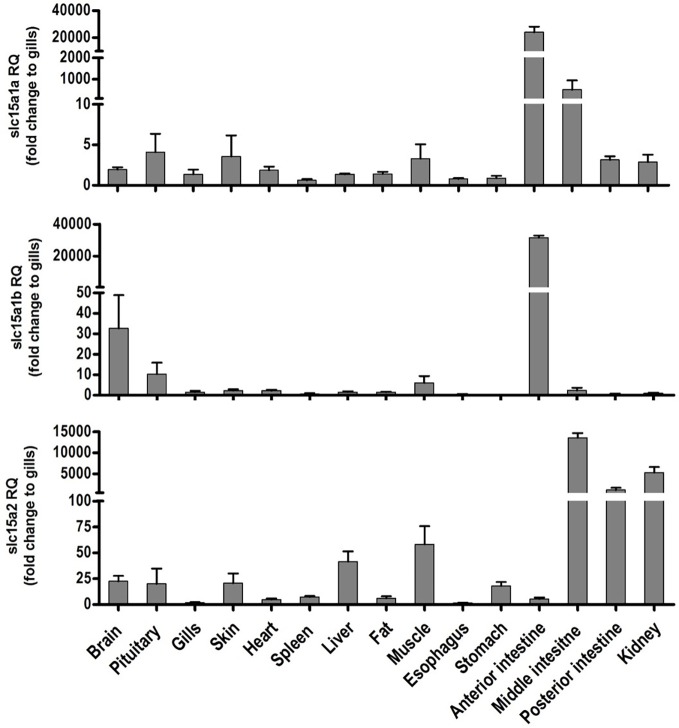
Relative expression of *slc15a1a, slc15a1b*, and *slc15a2* in different tissue of the adult Mozambique tilapia. The bars represent the average fold change of the tissue compare to the gills.

**Figure 2:**
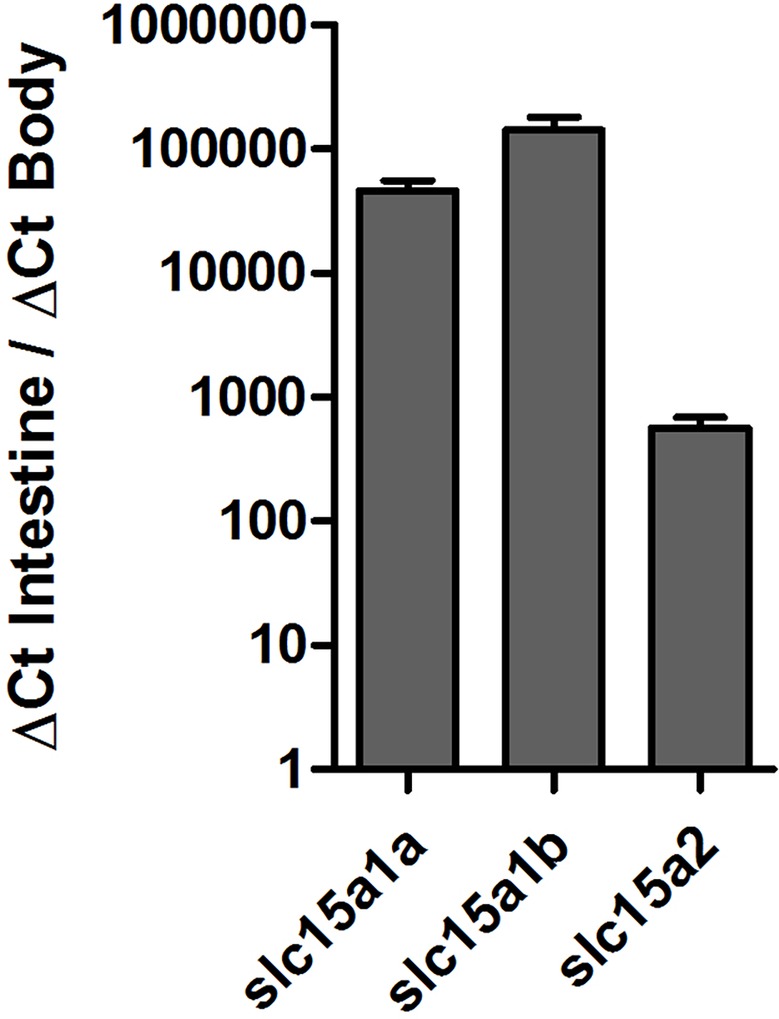
Expression of *slc15a1a, slc15a1b*, and *slc15a2* in the gastrointestinal tract in comparison to the body, in Mozambique tilapia larvae (9 dpf).

Tissue distribution of the PepT transcripts presented high abundance of the PepT transcripts in the intestinal segments (Figure 1). PepT1 variants were the highest in the AI segment (*p* < 0.0001), while PepT2 expression was detected in the MI and PI segments. PepT2 was also found to express in the kidney but in lower level compare to the MI segment (*p* < 0.0001). The expression level in the rest of the tissues was significantly lower (*p* < 0.0001) than in the intestine (Figure 1). Overall, the intestine and kidney were found to be the main tissues expressing the PepT variants. The qPCR analysis of dissected larvae revealed that the GI tract had significantly higher expression (*p* < 0.0001) of the PepT variants in comparison to the larvae body. The ratio between expression levels in the GI tract and the larvae body was approximately 565 for PepT2, 46,000 for PepT1a, and 140,000 for PepT1b transcripts (Figure 2).

**Figure 3:**
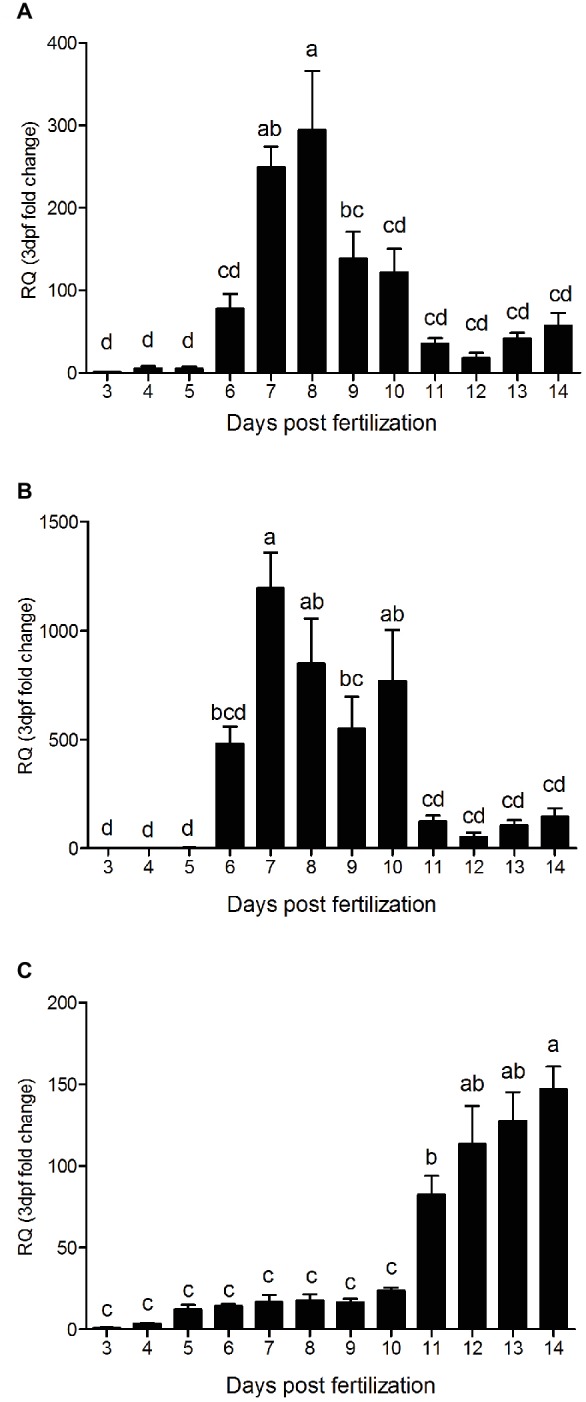
Relative expression of *slc15a1a* **(A)**, *slc15a1b* **(B)**, and *slc15a2* **(C)**, along 12 days post fertilization (3–14 dpf). Expression presented as fold change relative to the 3 dpf time point. Different letters indicate significant difference between days post fertilization.

The qPCR analysis of all three PepT variants revealed major differences between the expression patterns of the PepT isoforms (Figure 3). Both PepT1a and PepT1b relative expression showed significantly elevated expression at 7 dpf, followed by a decrease until 11 dpf. PepT2 transcript expression showed a different pattern from the other two, with a complementary trend. PepT2 expression did not change significantly between 3 and 10 dpf, followed by an elevation of expression at 11 dpf that was sustained until the end of sampling, at 14 dpf.

**Figure 4:**
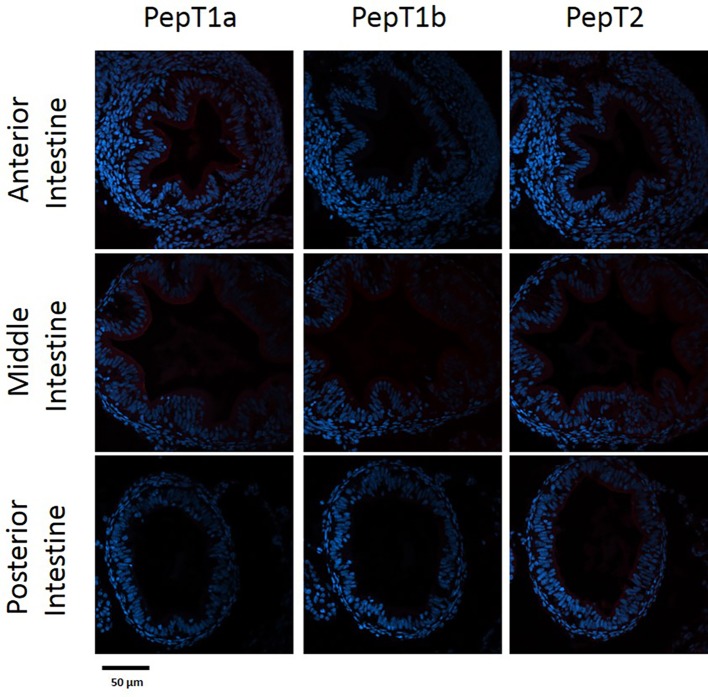
Immunofluorescence staining of cross sections of 7 dpf larvae with rabbit anti PepT (different variants) (red) and with Dapi for nuclei staining (blue). Scale bar 50 μm.

**Figure 5:**
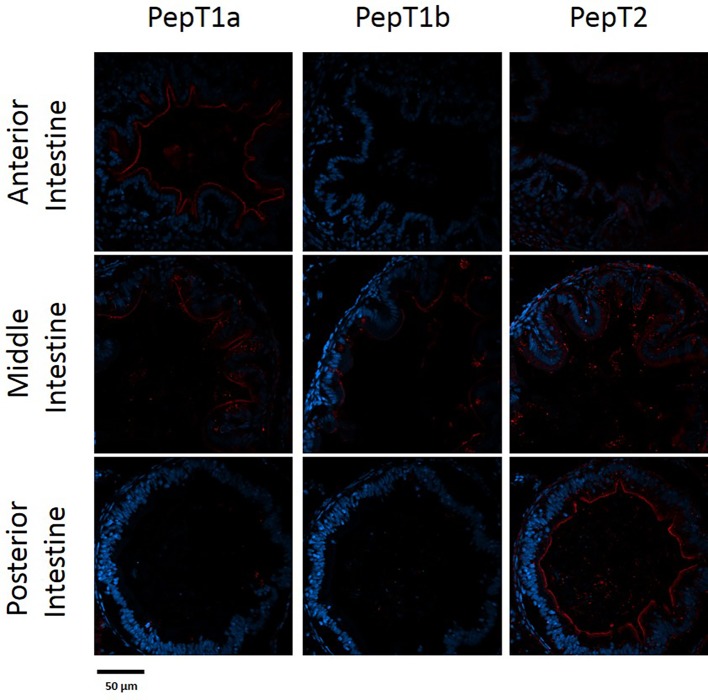
Immunofluorescence staining of cross sections of 14 dpf larvae with rabbit anti PepT (different variants) (red) and with Dapi for nuclei staining (blue). Scale bar 50 μm.

**Figure 6:**
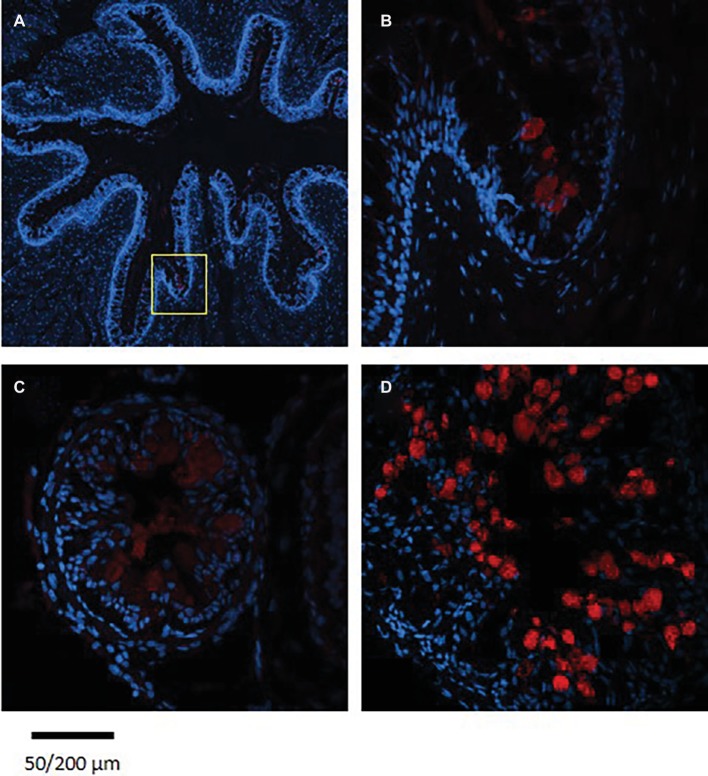
Immunofluorescence staining of cross sections of 7 dpf **(A)**, 14 dpf **(B)** larvae, and adult esophagus **(C,D)** with rabbit anti PepT (different variants) (red) and with Dapi for nuclei staining (blue). Scale bar 200 μm **(A)** and 50 μm **(B–D)**.

The staining of the cross sections revealed a section specific protein expression. PepT1a and PepT1b differed in their expression along the intestinal segments, with PepT1a staining in the esophagus and anterior to middle intestine and PepT1b expression starting only around the middle intestine. On the other hand, PepT2 staining was detectible only at the distal sections of the intestine (Figures 4, 5). There was no staining in the stomach of the larvae (data not shown). The esophagus staining was specific to the basolateral membrane of the mucosal cells, and only with PepT1a (Figures 6A,B), while at the different intestinal sections, all three antibodies stained the apical membrane of the enterocytes.

When comparing day 7 and 14 dpf, there was no significant difference in the staining of each variant (Figures 4, 5). However, at day 6 post fertilization, the only detection of PepT1a was in the esophagus and the anterior segment of the intestine (data not shown).

Staining of esophageal sections from an adult Mozambique tilapia exhibited more complex crypts and villi, with high number of goblet cells; however, only a few cells were stained with the PepT1a antibody. Although seen in a much lower number of esophageal goblet cells, the staining in the adult resemble to the one found in the larvae, with the entire cell stained (Figure 6).

## Discussion

Larvae differ greatly from the adults in various aspects (Dabrowski, 1984). Compared to the fully developed intestine of the adult fish, the larvae intestine is much less complexed (Govoni et al., 1986; Kolkovski, 2001). Along the intestine of adult fish, there are changes in various parameters of the lumen content, such as pH levels (Nikolopoulou et al., 2011), ion concentrations [reviewed by Marshall and Grosell (2006)], nutrients content (Orozco et al., 2018), and microbiome composition (Hallali et al., 2018). In line with these changes, differences between the intestinal sections have been shown in their morphology [reviewed by Wilson and Castro (2010)] and genes expression levels (Rimoldi et al., 2015; Ronkin et al., 2015; Nitzan et al., 2016; Con et al., 2017; Orozco et al., 2018). PepTs early expression in the Mozambique tilapia larvae is compatible with previous reports on early expression of protein digestion and absorption molecular systems. With the much higher PepTs expression in the larvae GI tract compared to the rest of the body (several orders of magnitude), we can safely consider the qPCR analysis of the whole larva as representative of the GI tract. In this study, PepT1 variants were detectable already from 3 dpf, with a significant increase at 6–7 dpf. This corresponds to the findings reported by Verri et al. (2003) for zebrafish embryos, showing detectable and increasing expression from 3 dpf. Our findings show a trend of steady increase of PepT2 expression from 11 dpf. In zebrafish, Romano et al. (2006) showed a steady expression for PepT2 from 3 dpf. In addition to these correlations, in Mozambique tilapia larvae, expression of trypsinogen and chymotrypsinogen was detectable from 1 day post hatching (Lo and Weng, 2006), which correspond to 6 dpf in our study. Trypsin was found to interact with the PepTs extracellular domain (Beale et al., 2015); thus, there is further support for the functionality of these small peptide absorption systems in early developmental stages.

PepT1 is a high capacity/low affinity transporter, fit to efficiently absorb at high substrate concentrations, while PepT2 is a low capacity/high affinity transporter, fit to efficiently absorb at low substrate concentrations. Indeed, Orozco et al. (2018) showed that protein contents decrease along the intestine during feed digestion and absorption. Corresponding to their kinetics differences, in the adult fish, PepT1 variants are highly expressed in proximal intestinal sections and PepT2 expressed in distal intestinal sections (Con et al., 2017). In addition, PepTs expression was shown to be affected by feed availability (Terova et al., 2009; Koven and Schulte, 2012; Tian et al., 2015). In line with the pattern observed in the adult fish, the immunofluorescence analysis demonstrated differential localization of the three PepT isoforms in the Mozambique tilapia pre-feeding larva. These findings indicate specialization of intestinal sections in the primary intestine, prior to exogenous feeding, and may suggest that these transporters are regulated by an additional mechanism, separate from exogenous feed availability.

The real-time analysis revealed that in parallel to the segments effect on the expression of these three variants, the expression was also affected by time points during this developmental period. The two PepT1 variants showed a similar expression pattern along the time course of the larval development, with a significant increase in expression levels at 6–7 dpf, while PepT2 showed an increase in expression levels only at 11 dpf, the same time point when there was a decrease in PepT1s expression. Such a complementary pattern, maintaining high expression of peptide transporters along larval development, implies on either a change in nutrients availability, or that these transporters have some different, unknown, roles during larval development. Nonetheless, the nutritional and developmental significance of intestinal nutrient absorbance for the larva, need further study.

A new observation was detected in the current research when examining the PepT1 variants. While in the adult Mozambique tilapia, PepT1a and Pept1b are expressed and localized together in the anterior and middle intestine, as shown in the tissue distribution analysis and in our previous work (Con et al., 2017), in the larvae these two variants are separated in the anterior intestine. PepT1a was found to express at the beginning of the GI tract, with strong expression in esophageal goblet cells and in the apical membrane of the enterocytes in the anterior and middle intestine. PepT1b protein expression on the other hand, started only in the middle intestine. Until now, intestinal co-expression of these two variants were only reported in adult tilapia (Huang et al., 2015; Con et al., 2017; Chourasia et al., 2018), Asian weather loach (*Misgurnus anguillicaudatus*) (Gonçalves et al., 2007), killifish (*Fundulus heteroclitus*) (Bucking and Schulte, 2012), and European sea bass (Kokou et al., 2019). These two variants, found in numerous fish species, resulted from the teleost-specific whole-genome duplication (Gonçalves et al., 2007; Con et al., 2017). The conservation of two paralogous genes through evolution indicates on functional difference between them. To our knowledge, this is the first time that a clear localization difference was found between PepT1a and PepT1b.

Immunofluorescence localization of the PepT1a in the esophagus was unexpected, as PepT1 expression was reported mainly in the intestine (Verri et al., 2003; Rønnestad et al., 2007; Terova et al., 2013), while the few studies that examined the esophagus did not detect PepT expression (Amberg et al., 2008; Ahn et al., 2013; Orozco et al., 2017). Two of these studies analyzed the PepT1b variant (Amberg et al., 2008; Ahn et al., 2013), which did not express in the esophagus also in our study. The tissue distribution analysis in the adult fish showed similar results to Orozco et al. (2017). However, immunofluorescence staining of adult and larvae esophagus showed a great difference in the stained goblet cells. The low abundance of stained cells in the adult tissue correlates with the low transcripts expression. Thus, this localization of PepT1a is a novel finding regarding differences between the larval and adult phenotypes.

In fish, the esophagus has an important role in maintaining salt and water balance. It has been shown that the desalination of seawater starts at the esophagus, which exhibit a low membrane permeability to water together with active and passive absorption of ions [reviewed by Grosell (2006)]. There are numerous goblet cells scattered throughout the esophagus epithelial, and it has been suggested that there are two goblet cells population differing by their maturation stage (Abaurrea-Equisoain and Ostos-Garrido, 1996). These cells participate in mucosa secretion in the GI tract. The mucosa plays a role in nutrients absorption and protection against pathogens (Bakke et al., 2010a). The expression of PepT1a in the goblet cells was unexpected and to the best of our knowledge, was never reported in any organism. Further investigation is needed in order to understand if this transporter participates in the mucosa production and/or secretion in the Mozambique tilapia larvae.

The localization of PepTs on the apical membrane of the enterocytes, as seen in the immunofluorescence staining, demonstrates that the intestine can uptake peptides even in the pre-feeding larvae. Our results indicate that in addition to the yolk sac absorption there might be another source of nutrients supplementation, even at early developmental stages. These nutrients might be of exogenous (passively driven into the larva gastrointestinal tract), endogenous, or of microbial source. This hypothesis, as well as the nutritional and developmental significance of intestinal nutrient absorbance for the larva, needs further study.

Many studies have shown the importance and effects of levels, and source and forms of protein in the feed on adult fish growth and physiology (Abdelghany, 2003; Failla et al., 2006; Ostaszewska et al., 2010; Kumar et al., 2012; Goosen et al., 2014). Zambonino Infante et al. (1997) showed that replacement of 20% of the fish meal in the fish diet with fish meal hydrolysate (75% di-tri peptide) significantly improved growth performance, increased proteolytic capacity of the pancreas, and affected intestinal enzymes activity in European sea bass larvae. In common carp (*Cyprinus carpio*), the addition of small peptides to the feed, increased the expression of PepT transcript and the abundance of cholecystokinin (CCK) secreting cells in the intestine (Ostaszewska et al., 2010). In mammalians primary tissue culture, it has been shown that the activity of PepT1 evoked the glucagon-like peptide-1 (GLP-1) secretion, depending on the activity of L-type Ca^2+^ channels (Diakogiannaki et al., 2013). These evidences for the participation of PepT activity in regulatory processes, together with the reports of the early expression in fish larvae, before full yolk absorption, raise the possibility that small peptides and PepT activity have some regulatory role in larval development and that the effects of small peptides on larvae development are mediated by PepTs activity in the enterocytes.

In summary, our results show that all three PepT variants are expressed in the intestine of the Mozambique tilapia during the larval development period, long before the onset of independent-active eating. The results also exhibit a difference between PepT1a and PepT1b protein expression. To our knowledge, this is the first evidence of the expression of all three PepTs in the intestine of fish larva. The early and section specific expression of these important nutrient transporters opens further questions regarding their role in the early larval stages.

## Data Availability

All datasets generated for this study are included in the manuscript and/or the supplementary files.

## Ethics Statement

This study was approved by the Agricultural Research Organization Committee for Ethics in Experimental Animal Use, and was carried out in compliance with the current laws governing biological research in Israel (Approval number: IL-650/15).

## Author Contributions

PC and AC conceived and designed the experiments. PC, TN, and TS bred, sampled and handled the fish. PC performed the microsurgery, gene expression, and immunofluorescence analyses. AC and SH secured funding and supervised the project. PC and AC wrote the manuscript.

### Conflict of Interest Statement

The authors declare that the research was conducted in the absence of any commercial or financial relationships that could be construed as a potential conflict of interest.
